# An Examination of the Feasibility of Detecting Cocaine Use Using Smartwatches

**DOI:** 10.3389/fpsyt.2021.674691

**Published:** 2021-06-24

**Authors:** Emre Ertin, Nithin Sugavanam, August F. Holtyn, Kenzie L. Preston, Jeremiah W. Bertz, Lisa A. Marsch, Bethany McLeman, Dikla Shmueli-Blumberg, Julia Collins, Jacqueline S. King, Jennifer McCormack, Udi E. Ghitza

**Affiliations:** ^1^Dreese Lab, Department of Electrical and Computer Engineering, Ohio State University, Columbus, OH, United States; ^2^Center for Learning and Health, School of Medicine, Johns Hopkins University, Baltimore, MD, United States; ^3^National Institute on Drug Abuse Intramural Research Program, National Institutes of Health, Baltimore, MD, United States; ^4^Center for Technology and Behavioral Health, Geisel School of Medicine, Dartmouth College, Lebanon, NH, United States; ^5^The Emmes Company, Rockville, MD, United States; ^6^Center for the Clinical Trials Network, National Institute on Drug Abuse, National Institutes of Health, Bethesda, MD, United States

**Keywords:** cocaine, measurement, clinical trials, mobile sensing, photoplethysmography

## Abstract

As digital technology increasingly informs clinical trials, novel ways to collect study data in the natural field setting have the potential to enhance the richness of research data. Cocaine use in clinical trials is usually collected *via* self-report and/or urine drug screen results, both of which have limitations. This article examines the feasibility of developing a wrist-worn device that can detect sufficient physiological data (i.e., heart rate and heart rate variability) to detect cocaine use. This study aimed to develop a wrist-worn device that can be used in the natural field setting among people who use cocaine to collect reliable data (determined by data yield, device wearability, and data quality) that is less obtrusive than chest-based devices used in prior research. The study also aimed to further develop a cocaine use detection algorithm used in previous research with an electrocardiogram on a chestband by adapting it to a photoplethysmography sensor on the wrist-worn device which is more prone to motion artifacts. Results indicate that wrist-based heart rate data collection is feasible and can provide higher data yield than chest-based sensors, as wrist-based devices were also more comfortable and affected participants' daily lives less often than chest-based sensors. When properly worn, wrist-based sensors produced similar quality of heart rate and heart rate variability features to chest-based sensors and matched their performance in automated detection of cocaine use events.

**Clinical Trial Registration:** www.ClinicalTrials.gov, identifier: NCT02915341.

## Introduction

Digital health technologies are changing the way we conduct research (1). Collecting data *via* digital devices [a.k.a., mobile health (mHealth)] in the natural field setting in which research participants make health choices presents an opportunity to collect granular data as individuals live their daily lives. These ecologically valid data may enhance the richness of research data, without excessive intrusion by researchers or strict reliance on participant recall.

Cocaine use detection in clinical trials is usually measured *via* self-report and/or urine drug screens. Urine drug screens (UDS) are considered the “gold standard” of use detection for many substances but are intrusive, subject to attendance at a research visit, and may be susceptible to adulteration. Participant self-reported use of cocaine can be inaccurate and unverifiable if not collected alongside other measures such as UDS. Passively sensing physiological data *via* wearable digital health technologies (e.g., heart rate and heart rate variability) may offer a potential avenue to collect objective data that can better inform the detection of use events and be unobtrusive and convenient for both participants and researchers.

Cocaine has a half-life of 1–2 h (2), and the onset of its effects vary by route of administration. The cardiovascular effects of cocaine use are acute. Cocaine induces heart rate elevation; Koenig et. al. and Vongpatanasin et. al. show that after cocaine intake, the heart rate variability measures such as high frequency (HF) oscillations, standard deviation of interbeat intervals from which artifacts have been removed (SDNN), and root mean square of successive interbeat interval differences between all successive heartbeats (RMSSD) decreased considerably while the mean heart rate increased (3, 4). This cocaine-induced heart rate elevation can be detected *via* sensors (5). Recent studies have demonstrated the feasibility of wearable sensors for illicit drug use detection in lab settings *via* electrocardiogram (ECG) sensors (6) but have identified several limitations for use in clinical trials in the natural field setting (7). These challenges include the limited ecological validity of data collected in lab studies, the qualitatively different samples of individuals who participate in lab vs. field studies, and the use of measurement devices (e.g., chestbands) that are not convenient or acceptable to participants to use in their daily lives.

Detection of cocaine use *via* wrist-worn devices could become an innovative method of leveraging convenient technology to aid in clinical trials that measure cocaine use. Additionally, current standard methods of detection (i.e., self-report or UDS) lack the temporal precision needed to identify antecedents and precipitants to cocaine use potentially available in continuous sensor data. Using mHealth to detect cocaine use in research could also provide critical information for the delivery of interventions in real time, or Just-in-Time Adaptive Interventions (6, 8).

Previous studies suggest that it is possible to collect high-quality, high-yield sensor data in the natural field setting with ECG-based sensor devices. Additional work is needed to minimize participant burden and maximize acceptability. Those sensors were worn around the chest and approximately one-third of participants considered them uncomfortable and reported that the devices made them feel self-conscious (9). Less obtrusive devices (such as wrist-worn sensors) may increase the collection of objective cocaine use detection data in the natural field setting. Heart rate detection in smartwatches is based on reflectance-based photoplethysmography (PPG) sensors. However, detection of interbeat intervals from PPG on a wrist-worn device is a challenging task, because weak signals are contaminated by the large motion artifacts caused by user's movement, which create noisy data that can infer false or mis-detected beats. Further, many of the techniques used in commercial smartwatches (10) target average heart rate *via* short-term recordings (~1 min). These techniques are not suited to provide interbeat intervals required for the application of advanced computational models needed to infer health states such as cocaine use events. It would be necessary to sample all sensor pairs at all times, but this can lead to high power consumption and short lifetime of the sensor battery. Commercially available smartwatch batteries last at most 10–11 h per charge with continuous sensor sampling, which is required for accurate interbeat interval monitoring. Such limited battery life limits the amount of time the device is available to collect data and increases participant burden with frequent recharging. In addition, most commercial offerings limit access to raw data and only provide heart rate information averaged over fixed small time segments, limiting their use in higher layer inference problems such as stress and drug use detection.

To refine mobile sensor technology to detect cardiac interbeat interval and physical activity data on a wrist-worn device that can be used to detect cocaine use in a field setting, sensing must be optimized to collect sufficient data and maintain needed battery life to properly collect the necessary amount of data.

This study built on previous work using a wireless physiological chest-based monitoring suite called AutoSense (11) to build a wrist-worn device and characterize the feasibility of using it to collect reliable interbeat interval and physical activity data in a field setting using PPG sensors. The AutoSense chest sensor has been previously used for in-the-field detection of cocaine use, as described above (5, 9); various members of the current study team were also part of this formative work with the AutoSense chestband.

In this study, the research team explored the use of wrist-worn PPG-sensing devices developed in the present study named as MotionSense HRV (12), paired with measurements from AutoSense chest sensor, ecological momentary assessments (EMA), and standardized assessments of substance use to characterize the amount of high quality data that can be obtained from PPG sensors on a wrist-worn device, identify common failure scenarios, and understand participant wearability and usage patterns. Additionally, the research team collected UDS results and self-reported cocaine use to compare against the sensor data. This article reports on this study in two phases: a preliminary pilot study to develop and test the wrist-worn device itself, and a main study to determine the feasibility of using wrist-worn PPG sensors to collect reliable data (determined by data yield, device wearability, and data quality) in the natural field setting. Additionally, the main study also aimed to adapt and improve the computational model previously used with ECG sensors for detecting cocaine use from interbeat interval heart rate data to apply to data derived from PPG sensors. The computational model used with ECG sensors for detecting cocaine use has been described in detail previously, see (5).

## Methods

This study was conducted in two phases: A Phase 1 Pilot Study (Pilot) and a Phase 2 Main Study (Main). The study team expected to enroll 5 participants in the Pilot and an additional 20 participants in the Main. The Pilot was designed to develop a wrist-worn device, through iterations of the sensors and data collection software, which could collect the needed heart rate data to prepare for use with the Main participants.

### Participants and Procedures

This study was conducted within the National Drug Abuse Treatment Clinical Trials Network funded by the National Institute on Drug Abuse. Participants from both the Pilot and Main phases were recruited from the Center for Learning and Health in the Department of Psychiatry and Behavioral Sciences at the Johns Hopkins University School of Medicine in Baltimore, MD, between May 2017 and March 2018. Participants were unemployed cocaine-using adults who were enrolled in a concurrent study at the site, known as the parent study (National Institute on Drug Abuse, R01DA037314, PI: Silverman) (13). Participation was offered to individuals who were active in the Induction Period of the parent study (days 8–35, during which participants were not expected to abstain from drug use and were exposed to the same conditions) at the time of study intake and had provided a cocaine-positive urine sample in the week prior (based on thrice-weekly urine samples collected as a part of the parent study). For a detailed description of the Induction Period, see (13). Briefly, participants were offered access to paid job-skills training in a model therapeutic workplace during the Induction Period. Potential participants also had to be available to attend weekday study visits at the research site and to participate for the full study duration (up to 16 days). Potential participants were approached by research staff and informed about the opportunity to participate in this study. Prior to giving informed consent, participants were invited to try on the AutoSense chestband and wrist-worn devices in order to factor device comfortability into their consent decision. If they agreed, participants provided written informed consent in accordance with good clinical practice, including the Declaration of Helsinki. After providing informed consent, participants were trained in the proper use of the wrist-worn devices and the AutoSense devices (smartwatches, chestband, and study Android-based smartphone). Participants were compensated for the amount of time they wore each device, the number of EMAs they completed, and for returning all study devices at the end of their participation. All recruitment, enrollment, and compensation procedures were the same for both the Pilot and Main phases. This project was approved by the Johns Hopkins University School of Medicine Institutional Review Board. More detailed information related to the study design and procedures was published shortly after enrollment commenced (14).

#### Characteristics and Drug Use Monitoring

In the Pilot and Main phases of the study, baseline assessments occurred on either Monday, Wednesday, or Friday in order to coincide with participants' UDS collection dates from the parent study (UDS data abstracted for use in this study). At baseline, a battery of assessment instruments was conducted, including the PhenX Core Tier 1 measures (15), which captured items such as recent and lifetime substance use, quality of life, and other health indicators, and a 7-day Timeline Followback (16), which collected data on substance use within the previous week. In addition, contact information was verified from the parent study. Photos were taken of participants' wrists using the study smartphone, both with and without the smartwatches, to aid in the identification of factors that may influence the quality of data collected *via* the smartwatches. After the baseline assessment, participants were asked to wear the AutoSense devices for 14 days, respond to EMA on the study smartphone, and visit the research site every weekday during the 14-day period. EMAs included questions about substance use, including specifics related to use events (such as type of drug used and the number of hits in each event), and indicators of stress, and participants had an option to self-report use outside of the thrice daily random EMA prompts. Participants attended research check-ins on weekdays to ensure proper wear of the study sensors and use of the study smartphone to transmit data to the study team. At these weekday visits, a use-specific Timeline Followback collected data on all substance use episodes by hits (e.g., if a participant reported one use event with 3 hits of cocaine, 3 use events would be reported for that item) that occurred since the previous check-in; additionally, participants provided UDS samples for the parent study and the results of which were shared with the study team at closeout.

#### Ecological Momentary Assessment

As a part of the parent study, participants made real-time self-reports of their mood, stress level, activities, craving, and drug use on an electronic diary (smartphone) provided by the study. In the Pilot and Main phases of this study, participants were prompted three times per day on the smartphone, at random times during their waking hours, to self-report on their mood, stress level, activities, and degree of craving. Participants were also asked to initiate an entry on the smartphone whenever they used cocaine, heroin, or other opioids or stimulants outside of a medical context; felt craving for cocaine, heroin, or other opioids; or felt overwhelmed, anxious, or stressed. Data from these EMA procedures were abstracted from the parent study.

#### Ambulatory Physiological Monitoring

In the Pilot, participants wore the AutoSense chestband and two wrist-worn devices (one on each wrist) for 2 weeks to monitor ambulatory physiological data *via* passive sensing. The AutoSense chestband collected data from (1) two-lead ECG measurement of electrical activity from the heart, (2) a respiratory inductive plethysmography band for measurement of relative lung volume and breathing rate at the rib cage, and (3) a three-axis accelerometer to assess motion artifacts in the data and provide inferences about physical activity. This unit is small (1 inch by 2.5 inches) and includes a 750-milliamp-h battery that did not require recharging for 10+ days. Data were collected from this unit *via* streaming sensor measurements *via* wireless radio connection (Bluetooth) to a smartphone in real-time.

Also in the Pilot, one wrist-worn device was worn on each wrist to maximize data capture from the devices and to account for within-subject differences in wrist anatomy, dominant hand, and wearing style. In the event of device failure or another scenario where two smartwatches were not available for a given participant, the use of one smartwatch was acceptable until an alternate could be provided for the missing device. Each of these devices contained a 9-axis inertial measurement unit (IMU) sensor comprised of 3-axis accelerometer, 3-axis gyroscope and 3-axis magnetometer sensors for the detection of activity and gestures. Each also contained reflectance PPG sensors, which used passive LED light reflectance to detect pulse waveform characteristics from the radial artery. IMU and PPG sensor data were collected by the wrist-worn device and then transmitted using Bluetooth wireless radio to the study smartphone in real-time. These data were used to derive characteristics of cardiovascular physiology, including heart rate and heart rate variability, on which the cocaine detection model was applied and refined. Data yield was derived from both the AutoSense chestband and wrist-worn devices in order to characterize the data capture from each device.

Participants were trained at baseline to put on the AutoSense chestband and chest electrodes. At each weekday clinic visit, the placement of the chestband, electrodes, and wrist-worn devices was checked by study staff, and participants were asked whether any of the devices were causing problems. Participants were given extra electrodes in case of detachment.

In the Main, procedures for ambulatory physiological monitoring *via* the AutoSense chestband and wrist-worn devices were identical to the Pilot. As described in the section Results below, the wrist-worn devices themselves underwent modifications based on results from the Pilot, but the procedures outlined above continued through the Main.

#### AutoSense Usability Questionnaire

At the end of their participation, participants were asked to respond to an 18-item questionnaire that assessed the usability and acceptability of both the AutoSense chestband and wrist-worn devices. Thirteen of the questions were multiple choice, measuring items such as participant's comfort putting on and wearing the devices, and whether they encountered problems while wearing them, whereas the five remaining items were open-ended and asked about their general experiences.

### Wrist-Worn Device Development

**Figure 1:**
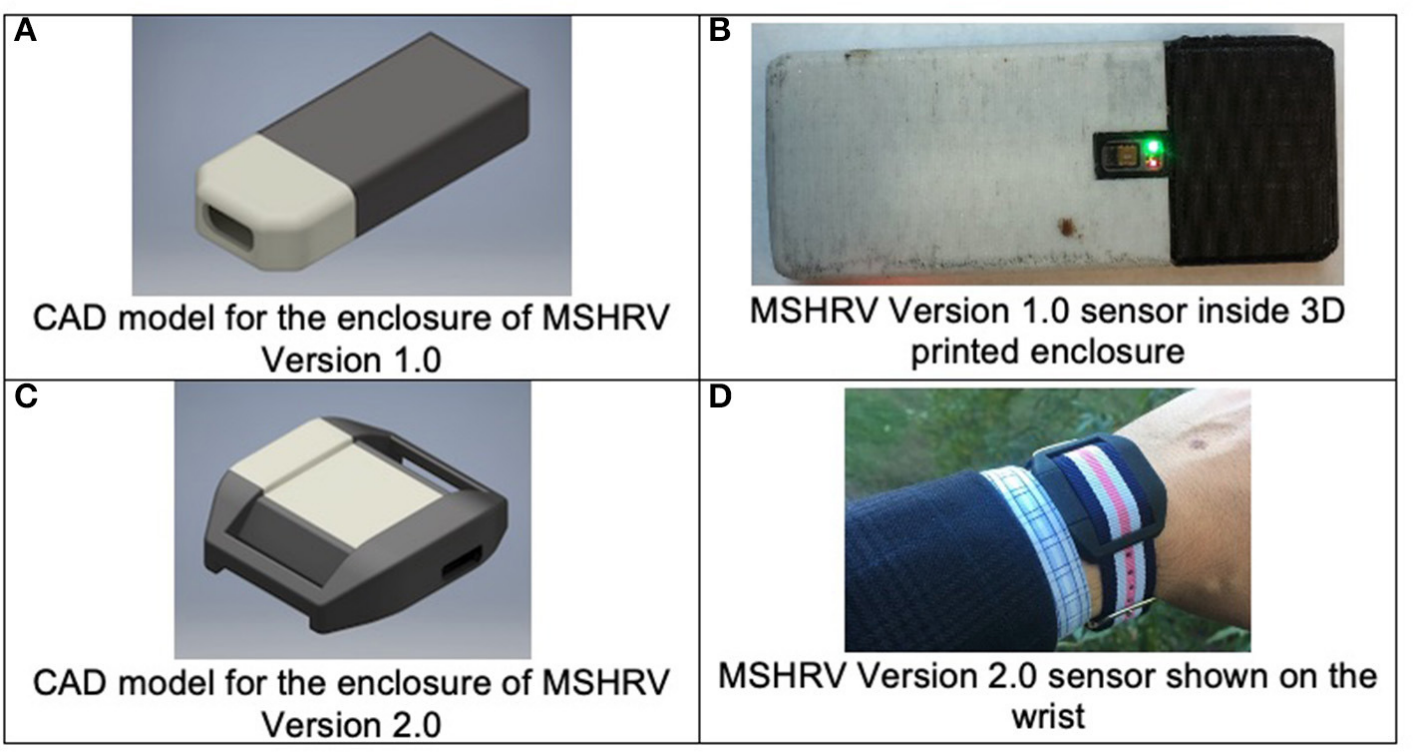
Wrist-worn sensor development. **(A,B)** Illustrate the version of the wrist-worn sensor used in the Phase 1 Pilot study. **(C,D)** Show the sensor used in the Phase 2 Main study.

Version 1.0 of the wrist-worn device, dubbed MotionSense Heart Rate Variability (MSHRV), was 42.5 mm long by 16.5 mm wide, resembling approximately the form factor of the commercial Fitbit One sensor; hence, Fitbit One wrist straps were used to begin the Pilot. Figures 1A,B show the computer-aided design image of the MSHRV Version 1.0 enclosure and the MSHRV Version 1.0 sensor in its 3D printed enclosure. The sensor included a 9-axis IMU sensor (accelerometer, gyroscope, and magnetometer) and a multispectral (red, green, infrared) PPG module from Maxim, and microcontroller from Nordic Semiconductor to perform inter-chip communication and wireless communication through Bluetooth Low Energy (BLE) to the smartphone. A custom 3D printed enclosure was designed and deployed.

The quality of the heart rate data collected by the sensor depends on the proximity of the sensor to the skin. For the first version, a commercial Fitbit One wrist strap was used to secure the sensor in place, resulting in a 2-mm gap between the PPG sensor and the user's skin. The Pilot revealed that this skin-sensor gap degraded the PPG signal quality significantly.

The first three participants recruited for the Pilot wore the first version of the wrist-worn devices (Version 1.0). Iterative improvements were made to the devices based on evaluation of the PPG data and participant-identified device failures, particularly reported issues with waterproofing of the internal sensor components (i.e., protection from sweat).

Problems identified *via* Pilot participants from the AutoSense Usability Questionnaire related to the fit of the device on the wrist were used to iteratively refine the wrist sensor devices for improved wearability and data collection, resulting in Version 2.0 of the wrist-worn devices. Version 2.0 utilized the same internal sensing components as Version 1.0 (e.g., microcontroller, LED/PPG sensor, inertial motion unit), but were refined to accommodate a more robust and comfortable enclosure, better waterproofing of the internal sensing components, and firmware updates to improve the LED intensity levels to improve data quality and yield compared to those of Version 1.0. Figures 1C,D show the MSHRV Version 2.0 sensor in its 3D printed enclosure. With the modified form factor of the PCB and the new enclosure design, the PPG sensor in Version 2.0 was flush with its enclosure and was in direct contact with the skin. This design change provided better heart rate signal quality compared to Version 1.0.

### Data Analysis

In the Pilot, descriptive statistics were used for the analyses of the usability questionnaire, participant characteristics and demographics, standard treatment and drug use monitoring, and EMA data. Participants were asked to engage in the study period for 16 days (14 days of device wear, with Day 1 for startup procedures and Day 16 for closeout procedures). The usability questionnaire and baseline characteristics and demographics were collected once, while urine drug screens and EMA random prompt data were expected to be collected more frequently (thrice weekly and thrice daily, respectively). The total number of outcome points per participant were calculated from these expected collection periods. Descriptive and inferential statistics were used to evaluate data yield (i.e., whether participants were wearing the devices and for what duration) and identify periods of time during which high quality data were collected by each sensor suite (i.e., the AutoSense ECG chestband and the PPG wrist-worn sensors). Data quality is defined as periods for which the heartbeat detection algorithm indicated continuous (1 min) segments of beats consistent with a human heart rate in the interval of 50–150 beats per min.

In the Main, the research team conducted the same analyses as in the Pilot. In addition, the cocaine-use detection algorithm was modified for use on data collected by the wrist-worn devices by preprocessing PPG signal to generate likely hypotheses for peak locations. Specifically, a probabilistic labeling algorithm developed previously by authors was used (17, 18) to generate likely sequences of heartbeats consistent with sensor data. These labels in turn were used to generate heart rate and heart rate variability measure features used in the cocaine detection algorithm (5). The study team used only events where sensors were worn and providing acceptable data quality to calculate the probability of detection. Detections were cross-referenced with EMA data to identify cocaine use events, and the cocaine detection algorithm was then run on those episodes (±3 h). Heart rate data for each episode was inspected manually to narrow down the cocaine episode timing when possible, as review of the data indicated that participants tended to only mark the timing approximately, usually rounding to the nearest 30 min. The research team secondarily compared data yield and data quality characteristics for the wrist-worn PPG sensors and the AutoSense ECG chest sensors. For the portions of the data where ECG and PPG signals were simultaneously available, the research team compared heart rate data from the two modalities using Bland Altman plots and linear correlation analysis (Pearson and robust statistics).

No analyses of stimulant use other than cocaine were conducted, as participants throughout the Main reported other stimulant use a total of 5 times (*via* EMA data), resulting in insufficient power to analyze these data within the current study.

## Results

### Participant Characteristics

#### Phase 1: Pilot Study

In the Pilot, six participants consented and enrolled out of an anticipated five (see Table 1). Half of the Pilot study population were male. On average, the age of Pilot participants was 51 (sd = 7.1) years, and none were Hispanic or Latino. Half of the participants self-reported as White and half as Black/African American. All six enrolled Pilot participants indicated that they used methadone in the 7 days prior to study enrollment. Pilot participants also indicated that they had used cocaine (1/6) or crack cocaine (5/6) in the 7 days prior to study enrollment, and the average number of days in which crack cocaine was used in that time was 3.3 days (sd = 2.34). Concomitant medications prescribed for enrolled Pilot participants included methadone, an Angiotensin Converting Enzyme (ACE) inhibitor, calcium channel blocker, anti-convulsant, proton pump inhibitor, antipsychotic, antidepressant, and a benzodiazepine.

**Table 1:** Participant demographics and characteristics.

**Characteristic**	**Pilot (*N* = 6)**	**Main (*N* = 14)**
Gender
Male	3 (50%)	10 (71.4%)
Female	3 (50%)	4 (28.6%)
Age [mean (std)]	51 (7.1)	47 (13)
Age
<18	0 (0%)	1 (7.1%)
18 <25	0 (0%)	1 (7.1%)
25 <35	0 (0%)	3 (21.4%)
35 <45	1 (17%)	4 (28.6%)
45 <55	4 (67%)	3 (21.4%)
55 <65	1 (17%)	2 (14.3%)
Ethnicity
Not hispanic or latino	6 (100%)	14 (100.0%)
Race
Black or African American	3 (50%)	9 (64.3%)
White	3 (50%)	5 (35.7%)
Education completed
Less than high school diploma	2 (33%)	4 (28.6%)
High school graduate	0 (0%)	6 (42.9%)
GED or equivalent	0 (0%)	1 (7.1%)
Some college, no degree	4 (67%)	3 (21.4%)
Marital status
Married	1 (17%)	2 (14.3%)
Divorced	1 (17%)	3 (21.4%)
Separated	0 (0%)	1 (7.1%)
Never married	4 (67%)	8 (57.1%)
Employment
Looking for work, unemployed	5 (83%)	11 (78.6%)
Disabled permanently or temporarily	1 (17%)	3 (21.4%)
Body mass index [mean (std)]	34 (10)	28 (6)
Most recent HIV test result
Negative	6 (100%)	12 (86%)
Positive	0 (0.0%)	2 (14%)
Smoked at least 100 cigarettes in lifetime
No	2 (33%)	1 (7%)
Yes	4 (67%)	13 (93%)
If yes, current smoking status
Every day	3 (75%)	11 (85%)
Some days	1 (25%)	2 (15%)

#### Phase 2: Main Study

In the Main, 20 participants consented and 19 enrolled out of an anticipated 20, with one participant not returning after signing consent.

Unfortunately, due to an error with the servers where sensor data provided by participants in the Main were stored, the study experienced a loss of some electronically stored data. Due to this loss of data, sensor data were available for a total of 14 Main participants. Of those for whom sensor data were available to analyze (*n* = 14, see Table 1): they averaged 47 (sd = 13) years of age; 10 were male; none were Hispanic or Latino; and the majority self-reported as Black/African American (9/14). All 14 Main participants indicated that they used methadone (13/14) or buprenorphine (1/14) in the 7 days prior to study enrollment. In the 7 days prior to study enrollment, 4 participants reported using cocaine on 3.8 days (sd = 2.5), and 11 reported using crack cocaine on 3.9 days (sd = 2.2). Concomitant medications for Main participants included methadone, buprenorphine, antidepressants, an antihypertensive, antineuropathic, calcium channel blocker, a blood thinner, HIV/AIDS antiretroviral, and a benzodiazepine.

### Primary Outcome Availability

#### Primary Outcome Availability in the Phase 1 Pilot Study

In the Pilot, data collection was completed with five participants each for 2 weeks (1 participant was withdrawn for a failure to wear the study devices), resulting in 10 expected weeks of data. As some primary outcome data were still collected from the withdrawn participant, those are represented here. During the Pilot, 39 of the 44 expected UDS (88.6% compliance) were collected; all 5 Pilot participants responded to at least one EMA random prompt, with an average (sd) random prompt completion rate of 87.6% (17.3%). Five of the six AutoSense Usability Questionnaire forms were collected, though only five were expected as one participant was withdrawn (100% compliance; Table 2).

**Table 2:** AutoSense usability questionnaire.

	**Main** **(*N* = 14)**
Number of participants who completed questionnaire	13
**In general, how easy is it to put on the AutoSense chest band?**	
Very easy	4 (30.8%)
Easy	9 (69.2%)
Difficult	0 (0%)
Very difficult	0 (0%)
**In general, how easy is it to put on the AutoSense smartwatch?**	
Very easy	8 (61.5%)
Easy	5 (38.5%)
Difficult	0 (0%)
Very difficult	0 (0%)
**In general, how easy is it to use the smartphone?**
Very easy	6 (46.2%)
Easy	7 (53.8%)
Difficult	0 (0%)
Very difficult	0 (0%)
**In general, how comfortable is it to wear the AutoSense chest band?**	
Very comfortable	2 (15.4%)
Comfortable	10 (76.9%)
Uncomfortable	1 (7.7%)
Very uncomfortable	0 (0%)
**In general, how comfortable is it to wear the AutoSense smartwatch?**	
Very comfortable	6 (46.2%)
Comfortable	7 (53.8%)
Uncomfortable	0 (0%)
Very uncomfortable	0 (0%)
**In general, how self-conscious did you feel while wearing the AutoSense chest band?**	
Very self-conscious	0 (0%)
Moderately self-conscious	2 (15.4%)
A little self-conscious	0 (0%)
Not self-conscious at all	11 (84.6%)
**In general, how self-conscious did you feel while wearing the AutoSense smartwatch?**	
Very self-conscious	0 (0%)
Moderately self-conscious	0 (0%)
A little self-conscious	0 (0%)
Not self-conscious at all	13 (100.0%)
**How often did you have problems with the AutoSense chest band?**	
Never	11 (84.6%)
One to two times during the whole week	1 (7.7%)
Three to four times during the whole week	1 (7.7%)
Five to six times during the whole week	0 (0%)
Every day	0 (0%)
**How often did you have problems with the AutoSense smartwatch?**	
Never	8 (61.5%)
One to two times during the whole week	2 (15.4%)
Three to four times during the whole week	2 (15.4%)
Five to six times during the whole week	0 (0%)
Every day	1 (7.7%)
**Did the AutoSense chest band affect your daily activities in any way?**	
No, I was able to do everything as usual.	9 (69.2%)
No, not really. I may have made a few adjustments to what I normally do.	4 (30.8%)
Yes, quite a bit and I had to change my routine.	0 (0%)
Yes, I totally changed my routine to accommodate the AutoSense.	0 (0%)
**Did the AutoSense smartwatch affect your daily activities in any way?**	
No, I was able to do everything as usual.	11 (84.6%)
No, not really. I may have made a few adjustments to what I normally do.	1 (7.7%)
Yes, quite a bit and I had to change my routine.	1 (7.7%)
Yes, I totally changed my routine to accommodate the AutoSense.	0 (0%)

As 6 participants were enrolled in the Pilot, it was anticipated that this would yield at least 36 individual cocaine use episodes (six episodes per participant were expected based on rates of cocaine use observed in the parent study). In actuality, at least 58 cocaine use events (doses or “hits”) were indicated by EMA data. Nearly 75% of UDSs from the Pilot were positive for cocaine. Additionally, one participant reported cocaine use, and five participants reported crack cocaine use during the study period *via* the Use-Specific Timeline Followback, totalling 171 episodes over the study period. Pilot study participants reported zero uses of other stimulants throughout the Pilot study period.

#### Primary Outcome Availability in the Phase 2 Main Study

During the Main, 89 of the 100 expected UDS (89.0% compliance) were collected; 92.9% of the AutoSense Usability Questionnaires were collected. All 14 participants reported substance use on the Use-Specific Timeline Followback throughout the study period, with four reporting cocaine use in 42 events and 11 reporting crack cocaine use in 140 events (indicating that at least one participant reported using both substances); and 11 of 14 (78.6%) participants responded to at least one EMA random prompt (see Table 3); for those 11 participants, the average (sd) random prompt completion rate was 73.3% (13.1%).

**Table 3:** Availability of primary outcome data in the main study.

	**Main (*N* = 14)**
**Urine drug screen^a^**	
Number collected	89
Number expected	100
Percent collected	89.0%
**AutoSense usability questionnaire**	
Number collected	13
Number expected	14
Percent collected	92.9%
**Use-specific timeline followback**	
Number of cocaine use episodes	42
Number of crack use episodes	140
**Ecological momentary assessment (EMA)**	
Completed at least one EMA entry *n* (%)	11 (78.6%)

a *The maximum number of UDS that a participant can contribute is no greater than the number of UDS expected per protocol*.

### Feasibility of Wrist-Worn Sensor Data Collection

#### Data Yield and Quality in the Phase 1 Pilot Study

During the Pilot, a heuristic motion-based algorithm was used to assess data yield. Motion, as detected *via* the IMU of the wrist sensors (containing accelerometer and gyroscope sensors), was used to detect whether participants were wearing the devices and for what duration. This algorithm employed a predetermined threshold to the accelerometer and gyroscope signals to indicate whether the sensors were being worn or not. Concurrently, Pilot participants were compensated for wearing the wrist-worn devices, which they did for 13.5 (sd = 5.06) h per day on average. Compensation was calculated *via* a data dashboard with real-time estimates of device wear. This real-time processing occurred on the smartphone device itself and used algorithms to evaluate data quality from the sensor data streams using the Cerebral Cortex platform developed in Hossain et al. (19), Hnat et al. (20), and Kumar et al. (21). Based on sensor patterns that deviated from an expected baseline, the algorithms evaluated whether the wrist sensor accelerometers were in motion (yielding an expectation of good data quality), not in motion (indicating a pattern of non-wear), or disconnected.

Similarly, algorithms were run on the smartphone to detect wear, non-wear, or disconnection of the AutoSense chest sensor suite for both the respiratory induction plethysmography (RIP) signal, as well as the ECG signal. These data quality metrics were computed approximately once every 3 s for each data stream (left- and right-wrist sensors, RIP, and ECG) and were uploaded to the backend of the data dashboard where these computations were expressed as hours of wear per day for compensation purposes. Research staff at the study site would access this dashboard at daily research visits, confirm participants' previous day's use of both the wrist-worn devices and chestband, and compensate them accordingly.

Although they were compensated for an average of 13.5 h of wear per day, an offline analysis of data yield from Pilot participants indicated that wrist-worn devices were only being worn on average 5.79 h per day. This offline analysis was based on PPG signal quality itself, as opposed to the real-time motion-based algorithm, which was based on motion alone, to compensate the participants *via* the Cerebral Cortex dashboard. This discrepancy indicated that the motion-based algorithm being used to define data quality and detect yield was confounded, perhaps by wearing the wrist sensors loosely or carrying the sensors in a bag/pocket instead of on the wrist, which will result in a motion signal being detected without a concurrent valid PPG signal. Research staff at the study site were also encouraged to routinely check Cerebral Cortex and discuss lags in data collection with participants during weekday study visits. Once the Pilot was complete, the research team made significant changes to the real time algorithm that detected data yield, integrating PPG sensing and skin contact detection (see Main Study results for further details). Participants from the Pilot wore the AutoSense chestband on average 6.50 h per day as determined by a data quality assessment algorithm tailored for ECG signals based on the expected signal morphology and periodicity. This ECG data yield algorithm assesses the morphology of the ECG signal in a fixed window (such as 3 s) through computing the outlier percentage, range, maximum, minimum, and other statistics from the window before rendering a decision.

#### Data Yield and Quality in the Phase 2 Main Study

Participants were expected to wear the sensor devices (both wrists and chest) for at least 10 h per day. On average, participants in the Main (*n* = 14) wore the left wrist sensor for 14.8 (sd = 3.1) h per day, the right wrist sensor for 14.1 (sd = 3.6) h per day, and the chest sensor for 16.2 (sd = 3.7) h per day for the 2-week study period. Though participants appear to have worn the chest sensor (ECG) longer, the quality of the data collected was superior in the wrist sensors (PPG); 68.7% of data collected from the left wrist sensor and 77.7% from the right wrist sensor were of acceptable quality, while only 34.4% of data were of acceptable quality. Acceptable quality in the data is defined as periods for which the heartbeat detection algorithm indicated continuous (1 min) segments of beats consistent with a human heart rate in the interval of 50–150 beats per min. Data quality metrics were calculated only for the periods that the sensor data were available. Additionally, data from the AutoSense Usability Questionnaire indicated that, compared to the chestband, participants thought the wrist sensors were very easy to put on (61.5 vs. 30.8%), very comfortable (46.2 vs. 15.4%), not self-conscious to wear (100 vs. 84.6%), and were better able to perform daily activities as usual (84.6 vs. 69.2%) vs. the chestband. However, participants more often reported never having problems with the chestband vs. wrist sensors (84.6 vs. 61.5%). These data indicate that yield, compliance, and sensor data quality were better in the wrist-worn sensors than the chestband sensors, though, as was somewhat expected, more problems were reported with the wrist-worn sensors developed in this study.

The study team also calculated correlation coefficients between ECG and PPG sensors, as the Pearson Correlation coefficient is known to be sensitive to outliers present in this type of data. Robust statistical estimates of the correlation coefficients (22, 23) were calculated (Table 4). The results show a wide range of variability (0.1–0.9) across subjects, indicating possible differences in how different participants wore the wrist sensors. The average yield of good quality ECG data is 5.6 h per day per participant. The correlation coefficients are computed on these intervals. The study team also observed that, in general, correlation coefficients were consistent across the left and right wristbands for the same subject, giving further credence to the hypothesis that incorrect sensor attachment or device wear is a major factor to low performance of the PPG sensor.

**Table 4:** Correlation coefficient between PPG and ECG sensors.

**Participant ID**	**Robust correlation coefficient between heartrate from ECG and left wrist PPG**	**Robust correlation coefficient between heartrate from ECG and right wrist PPG**	**Pearson correlation coefficient between heartrate from ECG and left wrist PPG**	**Pearson correlation coefficient between heartrate from ECG and right wrist PPG**
Participant 1	0.1	0.13	−0.1	−0.15
Participant 2	0.38	0.26	0.26	0.16
Participant 3	0.89	−0.06	0.21	−0.09
Participant 4	0.17	0.23	0.21	0.19
Participant 5	0.28	0.38	0.27	0.31
Participant 6	0.48	0.42	0.24	0.24
Participant 7	0.89	0.98	0.21	0.29
Participant 8	0.57	0.42	0.27	0.18
Participant 9	0.93	0.91	0.69	0.67
Participant 10	−0.08	−0.11	−0.07	−0.01
Participant 11	0.24	0.12	0.24	0.31
Participant 12	0.03	0.26	0.03	0.14
Participant 13	0.08	0.01	0.23	0.26
Participant 14	−0.1	−0.44	0.13	0.07

On average, female participants wore the PPG (wrist) sensors for about 1 h longer than males, and Black/African American participants wore both the PPG and ECG sensors nearly 2 h longer than White participants (Table 5).

**Table 5:** Data yield for PPG and ECG sensors by gender and race.

**Participants**	**Hours of ECG data: mean (sd)**	**Hours of PPG data: mean (sd)**
Female (*N* = 4)	5.30 (2.80)	12.50 (2.52)
Male (*N* = 10)	6.01 (3.95)	11.51 (2.99)
African American (*N* = 9)	6.53 (3.57)	12.56 (2.65)
White (*N* = 5)	4.50 (3.55)	10.43 (2.80)

**Figure 2:**
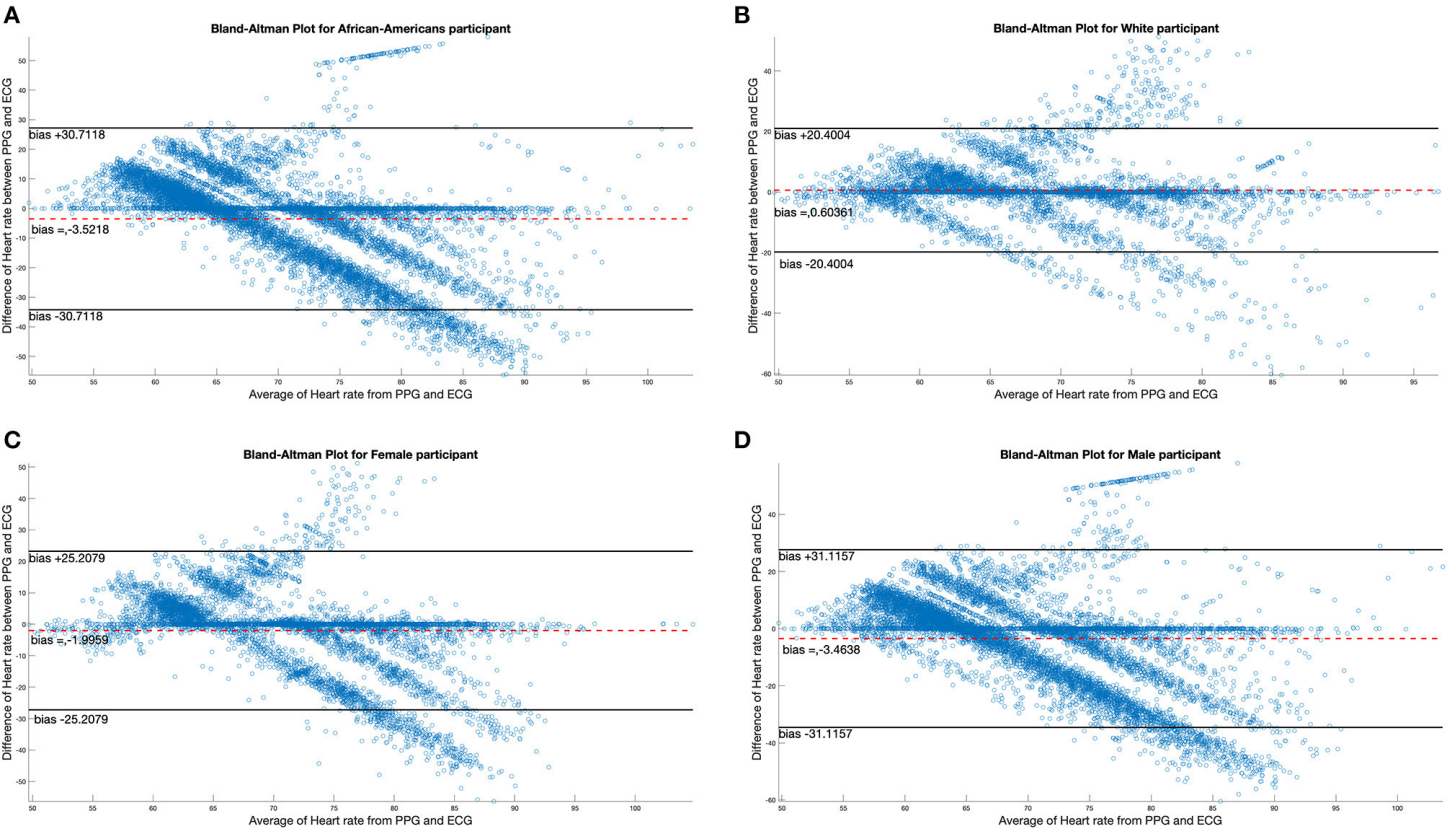
Bland-Altman plots comparing differences in ECG data by race and gender. **(A,B)** Illustrate the Bland-Altman plots comparing the ECG chest sensor and wrist sensor for African American participants and White participants. **(C,D)** Show the Bland-Altman plots comparing the ECG chest sensor and wrist sensor for female and male participants.

There were four female and 10 male participants; analyses of data quality and yield between these two groups (Table 5) found no statistically significant differences. Correlation between chestband and wrist-worn sensor readings were slightly higher for female participants (female *r* = 0.28; male *r* = 0.21). There were five White and nine African American participants; analyses of data quality and yield between these two groups (Table 5) found no statistically significant differences. The correlation between chestband and wrist-worn sensor readings was higher for White participants (African American *r* = 0.11; White *r* = 0.38). However, the ability to detect statistically significant differences may have been limited by the sample size. Figure 2 shows Bland-Altman plots comparing the ECG chest-sensor- and wrist-sensor-derived heart rates by race and gender. The participants are organized according to gender and race to illustrate the sensitivity of the wrist-worn sensors across these categories. The Y coordinate in the Bland-altman plot represents the difference between heart rate from PPG and heart rate from ECG. Heart rate from PPG falling below the heart rate from ECG produces negative error. Therefore, the Bland-altman plot shows that at higher heart rate the PPG sensor under-determines the heart rate estimate.

### Cocaine Use Detection

#### Cocaine Use Detection in the Phase 1 Pilot Study

As the main goal of the Pilot was to develop the device through iterations of the sensors and data collection software, no analysis of cocaine use detection was performed.

#### Cocaine Use Detection in the Phase 2 Main Study

Among the 14 participants for whom sensor data were available to analyze, a total of 58 cocaine use events were reported *via* EMA (cocaine alone: 37 events; cocaine with other drugs: 21 events) in the Main. Additionally, participants reported 42 cocaine and 140 crack cocaine use events throughout the study period *via* the Use-Specific Timeline Followback. Nearly all (98.8%) of the UDSs collected from these participants were positive for cocaine (Tables 6, 7). These data were meant to assist in identifying cocaine use events by sensor data (ECG and PPG). Among these 58 cocaine use events, the AutoSense chestband (ECG) was worn during 36 (62%), and at least one of the wrist-worn sensors was worn for 34 use events; 28 of these use events occurred while the participant was wearing both the chest and at least one wrist sensor (Table 8). The study team used only events in which sensors were worn and providing acceptable data quality to calculate the probability of detection. In general, the probability of detection with the PPG (wrist) sensor was higher for females {67%, [Confidence Interval (CI): 46, 83%]} compared to males [54%, (CI: 29, 78%)] and higher for White participants [86%, (CI: 62, 97%)] compared to Black/African American participants [45%, (CI: 26, 65%)] (Table 9).

**Table 6:** Summary of substance use in the main study.

	**Main (*N* = 14)**
**Any substance use over the study period^a^**	
**Tobacco**	
Number of participants who used substance	13
Number of episodes [mean (std)]	90.1 (47.93)
Total number of episodes over study period	1,171
**Alcohol**	
Number of participants who used substance	3
Number of episodes [mean (std)]	10.0 (3.46)
Total number of episodes over study period	30
**Cannabinoids/marijuana**	
Number of participants who used substance	5
Number of episodes [mean (std)]	1.0 (0.00)
Total number of episodes over study period	5
**Synthetic cannabinoids**	
Number of participants who used substance	0
Number of episodes [mean (std)]	0 (0)
Total number of episodes over study period	0
**Cocaine**	
Number of participants who used substance	4
Number of episodes [mean (std)]	10.5 (13.72)
Total number of episodes over study period	42
**Crack**	
Number of participants who used substance	11
Number of episodes [mean (std)]	12.7 (9.75)
Total number of episodes over study period	140
**Amphetamines**	
Number of participants who used substance	0
Number of episodes [mean (std)]	0 (0)
Total number of episodes over study period	0
**Methamphetamine**	
Number of participants who used substance	0
Number of episodes [mean (std)]	0 (0)
Total number of episodes over study period	0
**Heroin**	
Number of participants who used substance	8
Number of episodes [mean (std)]	7.6 (9.69)
Total number of episodes over study period	61
**Methadone**	
Number of participants who used substance	13
Number of episodes [mean (std)]	13.3 (3.33)
Total number of episodes over study period	173
**Buprenorphine**	
Number of participants who used substance	1
Number of episodes [mean (std)]	13.0 (0)
Total number of episodes over study period	13
**Other opioids**	
Number of participants who used substance	0
Number of episodes [mean (std)]	0 (0)
Total number of episodes over study period	0
**Hallucinogens**	
Number of participants who used substance	0
Number of episodes [mean (std)]	0 (0)
Total number of episodes over study period	0
**MDMA/ecstasy**	
Number of participants who used substance	0
Number of episodes [mean (std)]	0 (0)
Total number of episodes over study period	0
**Barbiturates**	
Number of participants who used substance	0
Number of episodes [mean (std)]	0 (0)
Total number of episodes over study period	0
**Benzodiazepines**	
Number of participants who used substance	4
Number of episodes [mean (std)]	7.8 (7.80)
Total number of episodes over study period	31
**Tranquilizers**	
Number of participants who used substance	1
Number of episodes [mean (std)]	4.0 (0)
Total number of episodes over study period	4
**Other sedatives and hypnotics**	
Number of participants who used substance	1
Number of episodes [mean (std)]	13.0 (0)
Total number of episodes over study period	13
**Inhalants/solvents**	
Number of participants who used substance	0
Number of episodes [mean (std)]	0 (0)
Total number of episodes over study period	0
**Other drug**	
Number of participants who used substance	5
Number of episodes [mean (std)]	20.0 (27.69)
Total number of episodes over study period	100

a *Substance use as reported on the Timeline Followback (TUS) form*.

**Table 7:** Summary of urine drug screens.

**Percentage of positive UDSs over the study period [mean (std)]**	**Main (*N* = 14)**
Benzodiazepines	34.5% (38.16)
Amphetamine	0% (0)
Marijuana	23.8% (30.89)
Methamphetamine	0% (0)
Cocaine	98.8% (4.45)
Oxycodone	0% (0)
Methadone	89.3% (28.95)
Opiates	41.1% (35.69)
Buprenorphine	10.7% (28.95)

**Table 8:** Cocaine use event detections in the main phase.

**Participant ID**	**Drug intake EMA report**	**PPG sensor present^a^**	**ECG sensor present**	**PPG and ECG sensor present^a^**	**Detections by PPG**	**Detections by ECG**	**Common detections**
Participant 1	5	5	5	5	1	4	1
Participant 2	0	0	0	0	0	0	0
Participant 3	2	2	1	1	2	1	1
Participant 4	1	1	1	1	1	1	1
Participant 5	17	10	15	8	9	5	4
Participant 6	6	5	6	5	4	4	4
Participant 7	0	0	0	0	0	0	0
Participant 8	5	1	0	0	0	0	0
Participant 9	3	2	2	2	1	1	1
Participant 10	5	1	1	1	0	1	0
Participant 11	0	0	0	0	0	0	0
Participant 12	2	1	1	1	1	0	0
Participant 13	10	5	3	3	1	0	0
Participant 14	2	1	1	1	1	1	1
**Total**	**58**	**34**	**36**	**28**	**21**	**18**	**13**

a *At least one wrist-worn sensor present*.

**Table 9:** Probability of detection of EMA events by gender and race.

**Participants**	**Probability of detection chestband (ECG)**	**Probability of detection wristband (PPG)**
Female (*N* = 4)	0.37	0.67
Male (*N* = 10)	0.75	0.54
African American (*N* = 9)	0.61	0.45
White (*N* = 5)	0.39	0.86

As summarized in Table 8, out of the 58 reported cocaine use events, there were 36 episodes in which chestband (ECG) sensor data were available, which resulted in 18 detections (probability of ECG-based detection 50.0%) of cocaine use indicated by the algorithm. This method focuses on the heart rate changes around the EMA-reported cocaine intake. Therefore, false positives are not feasible to evaluate. Data from at least one wrist (PPG) sensor were available in 34 episodes and resulted in 21 detections (probability of PPG-based detection 61.8%) of cocaine use. Finally, there were 13 cocaine use events detected by both sensors out of 28 use events when both sensors were available (probability of joint detection: 46.4%). While these detection probabilities are lower than the ones reported in previous studies of the AutoSense chestband (3), the PPG sensors achieved better probability of detection than the ECG sensors within this protocol. A careful visual inspection of the sensor data streams suggests that some self-reported use events may have been marked by the participants at the wrong time; in these reports, participants may have been “rounding” their use times to the nearest half-hour, rather than reporting the exact times. However, these events were kept in the analyses for uniform treatment, leading to overestimation of misdetection probability.

As the number of cocaine use events that were matched to known ground truth data collected (reported EMAs) was less than the expected number of use events required to refine the algorithm for use with PPG sensor data, the study team had to train the algorithm using estimations. Though participants' reported use of cocaine was as expected, the study team needed ~150 use events while participants were wearing all devices to refine the algorithm properly. As only 36 were available to analyze, the study team used a heart rate estimator for PPG signals trained with data from other pilot studies with the previously developed cocaine detection algorithm for the initial analysis.

## Discussion

This study developed a wrist-worn device that can be used in the natural field setting among people who use cocaine to collect reliable heart rate data that is less obtrusive than chest-based devices. Findings demonstrated that the quality of the data collected was superior in the wrist sensors (PPG) compared to the chest (ECG) sensors (68.7% of data collected from the left wrist sensor and 77.7% from the right wrist sensor were of acceptable quality, while only 34.4% from the chest sensor data were of acceptable quality). In terms of wearability, participants reported that the wrist sensors were easier to put on, more comfortable, less self-conscious to wear, and affected daily activities less often than the chest band. The study also further developed the cocaine use detection algorithm to identify cocaine use events from the wrist-worn PPG sensors. Results showed that when properly worn, wrist-worn PPG sensors can produce similar quality of heart rate and heart rate variability features to chest-worn ECG sensors that can be used for cocaine intake event detections. As described below, however, additional steps could be taken to more fully refine the cocaine use detection algorithm for wrist-based data collection.

Although results in this initial study evaluating the feasibility of heart rate detection *via* a wrist-worn device are promising, they also highlight several areas that require iterations before a system based on wrist-worn PPG sensors can be fielded to scale. First, in order to collect better quality data, the wrist-worn sensors have to be more robust to better account for different wear styles that affect contact with the skin, participant skin color which can interfere with certain LEDs, and motion artifacts to account for movement and other background noise not related to direct sensor contact (e.g., from being carried in a backpack). Based on the findings from this trial, the study team developed an improved version of the wrist-based sensor for use in another National Institutes of Health-funded study outside the scope of this project. This sensor featured a larger glass window with two green and two infrared LEDs and two photodiodes/receivers, resulting in a significantly larger signal-to-noise ratio and fewer motion artifacts.

Second, real-time indicators for participants to adjust the wrist sensor's fit or position are needed to improve the quality of sensor data being collected. The ability to notify the wearer that data quality is low and instruct the wearer regarding ways to adjust the wristband to improve data quality could markedly improve the data and cocaine-use event detection.

Third, improved self-reporting of drug intake events in real-time would improve robustness and the time resolution of the ground truth information that will produce training data to more fully refine the cocaine detection algorithm for wristband data. Though reporting *via* EMA has been fruitful, the results of this study indicate that the potential for participants to more conveniently and reliably report use with the wrist-worn device itself (e.g., double-tapping the wristband to mark a cocaine use event) could be very useful.

A few limitations of this study should be noted. First, complete data sets were not obtained from the originally intended sample of 25 participants, resulting in a smaller sample size than originally planned. Subsequent research will seek to obtain complete data sets from a larger sample of participants. Second, cocaine use events were inconsistently reported across the Timeline Followback and EMA entries, contributing to fewer identified use events that could be used to train the algorithm to detect use within PPG sensor data. Future studies can develop technology for in-the-moment reporting (i.e., double tapping the wrist-device). Third, no analyses of stimulant use other than cocaine use were conducted, as participants rarely used stimulants other than cocaine. Future research efforts can seek to recruit a larger sample of people who use both cocaine and other stimulants to allow for sufficient power to conduct such analyses.

### Future Directions of the Research

This line of research is innovative and at the early stages of its inception. There is a multitude of avenues in which a device such as this can be useful in clinical trials. Based on the promising results of this study, the next step in this line of research is to conduct a study with a larger sample size to increase the precision of cocaine use detection using unobtrusive PPG wrist sensors. The study would also utilize longer periods of assessment (potentially 12 weeks per participant) to increase the number of cocaine use episodes. This longer period of measurement may also enable us to understand better the utility of including this device as part of outcome measurement in future clinical intervention trials. Additional potential areas for extending research with this device include expanding into the detection of both stimulants and opioids, as analyzed in (20) by measuring peripheral capillary oxygen saturation (SpO_2_) to detect opioid use by reduced heart rate and blood oxygen saturation.

Additionally, the future of this research could include the analysis of whether there are different cardiovascular profiles associated with different routes of cocaine administration or with polydrug use, as well as whether polydrug use (vs. use of cocaine alone) affects the reliability of detecting cocaine use (24). Once the detection algorithm has been refined, it is also possible to study whether the algorithm can detect differences in the route of cocaine administration. This device and detection of cocaine use may also help to iteratively adapt treatment options to increase engagement and retention and improve patient-centered care. That is, the evolution of this line of research and measurement-based care could lead to the proactive monitoring and real-time detection of substance use, including polysubstance (e.g., stimulant and opioid) use, which may inform in-the-moment intervention delivery. It may allow for the identification of populations with differential risk profiles, which may inform tailored intervention delivery.

## Data Availability Statement

The raw data supporting the conclusions of this article will be made available by the authors, without undue reservation.

## Ethics Statement

The studies involving human participants were reviewed and approved by Johns Hopkins University School of Medicine Institutional Review Board. The patients/participants provided their written informed consent to participate in this study.

## Author Contributions

EE, AH, KP, LM, and UG developed the study protocol and oversaw the conduct of the study. KP oversaw data collection with participants. EE, NS, AH, JB, BM, JK, and JM analyzed the data. All authors discussed the results and contributed to the final manuscript.

## Conflict of Interest

DS-B, JC, JK, and JM were employed by the company The Emmes Company, LLC. The remaining authors declare that the research was conducted in the absence of any commercial or financial relationships that could be construed as a potential conflict of interest.
